# Unveiling Natural and Semisynthetic Acylated Flavonoids: Chemistry and Biological Actions in the Context of Molecular Docking

**DOI:** 10.3390/molecules27175501

**Published:** 2022-08-26

**Authors:** Dina M. El-Kersh, Rania F. Abou El-Ezz, Marwa Fouad, Mohamed A. Farag

**Affiliations:** 1 Department of Pharmacognosy, Faculty of Pharmacy, The British University in Egypt (BUE), Cairo 11837, Egypt; 2 Pharmacognosy Department, Faculty of Pharmacy, Misr International University, Cairo, Egypt; 3 Pharmaceutical Chemistry Department, Faculty of Pharmacy, Cairo University, Cairo 11562, Egypt; 4 Pharmaceutical Chemistry Department, School of Pharmacy, New Giza University, Newgiza, km 22 Cairo-Alexandria Desert Road, Cairo 12577, Egypt; 5 Pharmacognosy Department, College of Pharmacy, Cairo University, Kasr El Aini St., Cairo 11652, Egypt

**Keywords:** acylated flavonoids, medicinal plants, acetylcholinesterase, anti-diabetic, hepatoprotective

## Abstract

Acylated flavonoids are widely distributed natural metabolites in medicinal plants and foods with several health attributes. A large diversity of chemical structures of acylated flavonoids with interesting biological effects was reported from several plant species. Of these, 123 compounds with potential antimicrobial, antiparasitic, anti-inflammatory, anti-nociceptive, analgesic, and anti-complementary effects were selected from several databases including SCI-Finder, Scopus, Google Scholar, Science Direct, PubMed, and others. Some selected reported biologically active flavonoids were docked in the active binding sites of some natural enzymes, namely acetylcholinesterase, butyrylcholinesterase, α-amylase, α-glucosidase, aldose reductase, and HIV integrase, in an attempt to underline the key interactions that might be responsible for their biological activities.

## 1. Introduction

Flavonoids are secondary metabolites that are widely distributed in *planta*, and they are well recognized for their health benefits [1,2], viz., anticancer, antioxidant, anti-inflammatory, antiviral, and as neuro- and cardio-protective effects [3]. Flavonoids are phytochemicals comprising a benzopyrone ring bearing a phenolic or poly-phenolic group at different positions, classified based on their chemical structure, degree of unsaturation, and oxidation of carbon ring, *viz.*, anthoxanthins (flavanone and flavanol), flavanones, flavanonols, flavans, chalcones, anthocyanidins, and isoflavonoids [4]. Generally, the diversity among flavonoids’ classes arises from structural modifications on the basic skeleton, such as hydroxylation, methylation, acylation, and glycosylation, and further can affect the flavonoid’s physicochemical properties, stability, solubility, and bioavailability [2,5,6]. As per flavonoids, modifications of basic skeletons usually occur in planta through tailored enzymatic reactions.

**Figure 1:**
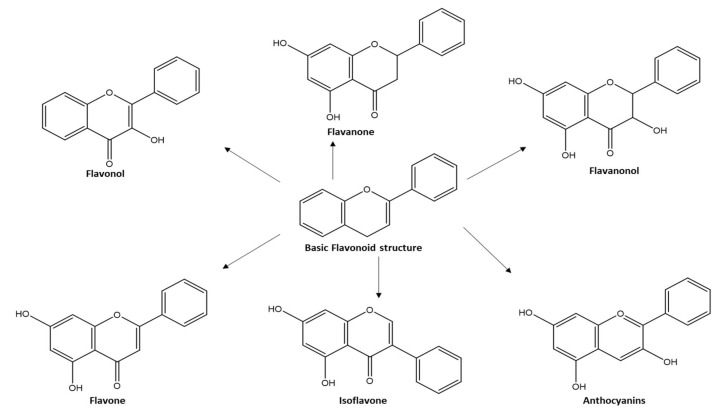
Chemical modifications on flavonoid structure.

A detailed analysis of plant metabolites showed the natural presence of acylated flavonoids in 14 plant species, mainly existing in the flavonol subclass. The interest in dietary flavonoids is attributed to their positive roles in various ailments, viz., cardiovascular risks, cancer, anti-inflammatory, enzymes inhibition, and antimicrobial and antiviral effects [1,7,8]. Although flavonoids are well recognized for their medicinal effects, their applications are rather limited due to restrictions in their solubility and stability, with the presence of numerous hydroxy groups that negatively impact their penetration inside lipophilic cell membranes or affect the binding to their cellular targets [1,7,8]. Chemical modifications of the flavonoid’s structure could thus strongly influence their biological activity as in Figure 1. The absence of a double bond at C2-C3, C3-OH group and C3’-C4’ dihydroxyl groups has a negative impact on the biological function of the flavonoid. The semi-synthetic acylated flavonoids should, thus, be carried out with a proper number, position, and types of acylation groups to ensure the maintenance of activity or its improvement [5].

The aim of this review is to introduce major biomedical activities of different natural acylated flavonoids and the impact of acylation on their biological activities (hepatoprotective, antidiabetic, neuroprotective, antioxidant, anticancer, and anti-inflammatory) in addition to a molecular docking study on the most documented biological activities to confirm features critical for efficacy in acylated flavonoids.

It is worth mentioning that the acylation of flavonoids with aromatic carboxylic acids enhances free radical scavenging activities [6,7] as well as UV protective effects and thermal and light stability. Likewise, acylated flavonoids show better insect repellent and pollen attractant effects [8]. Herein our report, we will introduce various semi-synthetic flavonoids, emphasizing the impact of semisynthetic and/or natural acylation on flavonoids and showing the correlation between acylation and the improvement of biological activities of the parent flavonoid by achieving improved solubility, stability, bioavailability, and lipophilic cell-membrane penetration [1,5,8].

## 2. Experimental Section

### Molecular Docking Study on Enzymes Inhibitory Compounds

From the Protein Data Bank (https://www.rcsb.org/), the X-ray crystal structures of acetylcholinesterase (PDB ID: 4EY7) [9] in complex with Donepezil, butyrylcholinesterase (PDB ID: 4BDS) [10]; in complex with Tacrine, α-amylase (PDB ID: 4W93) [11]; in complex with montbretin A, α-glucosidase (PDB ID: 2QMJ) [12]; in complex with Acarbose, aldose reductase (PDB ID: 3RX3) [13]; in complex with Sulindac and HIV integrase (PDB ID: 1QS4) [14]; and in complex with 1-(5-chloroindol-3-yl)-3-hydroxy-3-(2H-tetrazol-5-yl)-propenone were downloaded.

All the molecular modeling studies were carried out using Molecular Operating Environment (MOE, 2020.0901) software. Energy minimization was performed until an RMSD gradient of 0.05 kcal∙mol^−1^ Å^−1^ with MMFF94x forcefield was obtained, and then partial charges were automatically calculated. Protein preparation was carried out by removing water molecules and unnecessary ligands, protonating the protein by the 3D protocol in MOE using the QuickPrep default option in MOE and keeping one chain. Triangle Matcher placement methods and London dG scoring functions were used for the docking protocol, and the refinement of the produced poses was performed by using forcefields.

## 3. In Vitro Semi-Synthetic Acylation of Flavonoids and Their Main Actions

In this section, reports on the in vitro modification of flavonoids via the addition of fatty acids will be presented in a context relative to the flavonoids’ main actions. The most common acylation enzyme is lipase enzyme. Several types of lipases are capable of catalysing the acylation of flavonoid glycosides [1,15]. In a previous study [1], different organic solvents were used in the acylation of flavonoid molecules using *Candida antarctica* lipase B enzyme (CALB) such as the acylation of a citrus flavanone, hesperidin, with decanoic acid catalyzed by CALB. In another study [16], immobilized lipase enzymes obtained from *Candida antratica* were used for acylating rutin and esculin sugar residues with fatty acids. A higher yield of esterified flavonoids up to 70% was obtained upon using aliphatic fatty acids with a carbon number greater than 12 carbons. Spectroscopic analyses revealed that esterification was carried on a primary hydroxyl group of glucose moiety of esculin and on a 4’’’ hydroxyl group of rhamnose sugar of rutin. The author correlated the high acylation yield of esculin “being better acyl acceptor” than rutin to the presence of primary hydroxyl group in esculin glucoside structure, which is more reactive in esterification reactions; moreover, the bulkiness of the rutin structure influenced the acylation reaction’s yield negatively. Another factor that affects the acylation yield was lipase enzyme source, as it influences the type of esterification (for example, the lipase obtained from *Candida antarctica* esterifies the hydroxy groups of the sugar moieties), while lipase from *Pseudomonas cepacia* acylates the hydroxy groups of flavonoid aglycones. Different flavonoids were successfully acylated by lipase enzymes using decanoic acid as an acyl donor and hesperidin as an acceptor at a yield of 55% of hesperidin decanoate [1,5]. Another study [17] examined the regioselectivity of lipase enzyme on the esterification of rutin and isoquercetin via molecular modelling. Results showed that the aglycone part of both flavonoids was stabilized by the hydrogen bond, hydrophobic interactions, and localized at the entrance of the binding pocket. The sugar part was placed close to the bottom of the pocket, whereas acetylation occurred only at the primary 6′′-OH of glucose (isoquercetin) and secondary 4′′′-OH of rhamnose (rutin) [15]. The acylation of esculin and rutin sugars was examined by short carboxylic acids using carbohydrate esterase enzyme “acetyl esterase” in an aqueous medium. Esculin was efficiently converted to its acetate and propionate derivatives in a non-buffered aqueous medium. The conversion to acylated derivatives appeared to be the best with vinyl acetate and decreases with an increasing number of carbons in the carboxylic acid ester used as the acyl donor, rutin; due to its low solubility in water, it was successfully acylated only in 2-propanol, and vinyl acetate only worked as an acyl donor for rutin. Despite the bulkiness of the aglycone, the acceptor properties of the sugar moiety, such as glucosyl residues in esculin, was not affected, as the enzyme catalyzed transacetylation and transpropionation to the 3-position. The efficiency of the reaction decreased dramatically when using vinyl propionates instead of vinyl butyrate as the acyl donor [15].

Flavonoids are strong antioxidants, and improving their bioavailability through the enhancement of their lipophilic nature is expected to enhance its antioxidant action [18]. The investigation of the antioxidant activity of rutin and its fatty acid esters was expressed as a percentage of DPPH radical-scavenging activity compared to Butylated hydroxytoluene (BHT). Rutin fatty acid esters inhibited lipid peroxidation and was proved to be efficient DPPH-radical scavengers. The most potent inhibitors were rutin palmitate, rutin stearate, and rutin linolenate, with percentage inhibitions of 78.3%, 63.8%, and 60.7 %, respectively. Factors other than the lipophilicity influence antioxidant activities; i.e., the nature of the acyl donor related to the unsaturation of fatty acids in addition to the number of carbons could influence activities. The anticancer action of flavonoids was correlated with the inhibition of certain enzymes related to cell proliferation, differentiation, apoptosis, angiogenesis, and metastasis [19]. Esterification with long chains fatty acids (C_8_–C_18_) showed improved antitumor action. It should be noted that the anticancer activity was not only influenced by the drug’s lipophilic nature but also by the drug’s affinity to the cell receptors [20]. The anti-inflammatory mode of action of acylated flavonoids depends mainly on the inhibition of eicosanoids generating enzymes as well as the release of inflammatory mediators, i.e., histamine. Previous report on flavonoids, e.g., diosmetin, hesperetin, kaempferol, and astragalin, showed improved anti-inflammatory activities up to 30 times upon acylation [21]. Antimicrobial activities of several flavonoids improved by acylation due to the increased lipophilicity in addition to the increased stabilization of the phenol function [22].

In a synthetic trial, the effect of fatty acid carbon-chain length on the inhibition activity of 5α-reductase enzyme was assessed. The 5α-reductase enzyme is responsible for the conversion of testosterone to 5α dihydrotestosterone (DHT), and DHT is an androgen required for the development and function of the prostate gland. The 5α-reductase enzyme malfunction could lead to prostatic hyperplasia or even cancer. Epigallocatechin-3-gallate (EGCG) from green tea proved to have an inhibitory potential to 5α-reductase enzymes. The addition of a series of fatty acids to EGCG residue led to the semi-synthesis of epigallocatechin-3-O-palmitoleate (C16:0) with an IC_50_ of 0.53 μM, which was 12 times more potent than EGCG (IC_50_ = 6.29 μM). Increasing the lipophilicity of the molecule may improve its bioavailability, whereas inhibitory activities decreased with an increase in more than 16 carbon atoms. Unsaturated fatty acid acylation could slightly elevate its EGCG action compared to its saturated chain [23].

Another study on the synthetic acylation of long chain fatty acids with phloridizin and isoquercetin flavonoids was conducted to investigate their esters on tyrosinase enzyme inhibition. This enzyme has a role in browning plant tissues and human melanogenesis. The tyrosinase enzyme plays a role in dermatological ailments as hyperpigmentation and melanoma [24]. Regioselective enzyme acylation by lipase enzyme using acyl donors such as monosaturated and polyunsaturated fatty acids yielded less polar flavonoids. The α-linolenic acid ester of isoquercetin showed tyrosinase % inhibition in vitro at concentrations of 10, 100, and 1000 µM as 55.78, 78.77, and 85.55, respectively, whereas those by the docosahexanoic acid ester of phloridizin at the same previously mentioned concentrations demonstrated 25.7, 62.19, and 91.2%, respectively. Both α-linolenic acid esters of isoquercetin and the docosahexanoic acid ester of phloridizin were observed as the best inhibitors of tyrosinase enzymes, showing percentages of inhibition as follows: 25.71% at 10 µM, 62.09 at 100 µM, and 91.21% at a concentration of 1000 µM. The presence of multiple double bonds of fatty acids were found crucial for tyrosinase inhibition, whereas the length of the fatty acid chain should still be optimized to a certain number in order to avoid improper binding to the enzyme’s active sites [25].

Herein our review, we will present how natural acylated flavonoids, obtained from plant sources or functional foods, and their biological effects differ from their parent ones in context to flavonoids. Various biological effects are described, including hepatoprotective, anti-diabetic, enzyme inhibitors, cardioprotective activity, vasorelaxant and haematological activity, and miscellaneous activities (antifungal activity, wound healing activity, and anxiolytic activity).

## 4. Occurrence of Natural Acylated Flavonoids in Planta

*Brassica* vegetables have been well known for their richness in flavonoids, their anticancer roles, and their roles in the treatment of cardiovascular disorders due to their free radical scavenging activity. The majority of flavonoids detected in cauliflower included combinations of glycosylated kaempferol, as well as their mono- and diacylated derivatives mainly with sinapoyl, methoxy caffeic, caffeic, *p*-coumaric, and feruloyl moieties [26,27]. Isoflavone, viz., daidzein, genistein, and glycitien glucosides, in soybean naturally occurs as malonyl glucosides and is typical of legumes. Malonated glucosides ensure the stability, solubility, and transport properties for the vacuolar storage of these isoflavone conjugates as catalysed by (BAHD) family acyl transferase enzymes [28].

Anthocyanins represent a class of flavonoids responsible for the colour and organoleptic properties of different foods such as grapes and sweet potato [29,30]. Anthocyanins are considered to have potential in treating some human ailments such as cardiovascular disorders and have anticancer and anti-inflammatory roles since they are free radical scavenging agents [29,30,31]. Anthocyanins acylation could be conjugated with either aromatic moieties to sugar moieties in anthocyanins, viz., “*p*-coumaric, caffeic, sinapic, ferulic and gallic acids” or aliphatic moieties such as “malonic, acetic, malic and oxalic acids”, and these typically occur mainly on C3 and less on C6 or C4 of anthocyanins’ sugar structures [30]. Naturally acylated anthocyanins were detected in red wine grapes distributed in China [32]. Anthocyanins are responsible for the skin pigmentation of grapes berries, while acylated flavonols, which are bitter, aid in stabilizing the colour pigmentation of anthocyanins [32,33]. Different types of acylation were predicted in different grapes varieties and suggestive for different acylating enzyme activities. Interestingly, non-acylated anthocyanins are more enriched in grapes grown in cold climate regions. In contrast, regions characterized by having high temperature, humidity and low altitude produced grapes with higher levels of acylated anthocyanins. Anthocyanins acylated by ferulic acid were found in relatively high-altitude regions [32]. In Italy, a study on phenolics profiling 34 varieties of grapes in two consecutive years, 2006–2007 [34], revealed a diversity of acylated anthocyanins at the C6-glucose moiety by aliphatic “acetyl” and aromatic “coumaroyl and caffeoyl” moieties. It was observed that in some grape vintages, acylation with *p*-coumaroyl moiety was more predominant than that of caffeic acid and was in correlation with the prevalence of acyl transferase enzyme relative to *p*-coumarate moieties. It was also observed that the acylation level was lowered in cooler climates and in accordance with previous studies reported in grapes [32,35]. The profiling of anthocyanins in Korean grapes vintages using HPLC revealed that anthocyanins acylation was predominant with the *p*-coumaroyl moiety [31]. Acylated anthocyanin glucosides isolated from grapes skin were found to be more stable than their respective glucosides at high temperatures up to 100 °C, after which degradation occurred due to oxidation reactions [29]. It is worth mentioning that acylated anthocyanins possess more remarkable colouring pigmentation effects due to their stability to heat, light, and pH changes and in correlation with inter- and intra-molecular co-pigmentation and complex formation with metal ions. This could be utilized as natural food colorants, preferably more than synthetic ones that may cause adverse effects [30].

Anthocyanins composition in foliar sweet potato (*Ipomoea batatas* L.), identified using HPLC, revealed that the majority of anthocyanins belongs to the acylated cyanidin type rather than peonidin type, with mainly mono- and dicaffeic acids [36]. Cyanidin anthocyanins are more effective as they are antimutagenic, hepatoprotective, antidiabetic, and are antioxidants [36,37], while simultaneously posing as sweet potato leaves as functional foods for potential incorporation in nutraceuticals [36]. Another study on the roots of sweet potatoes (*Ipomoea batatas* L. Cv. Ayamurasaki) by HPLC-MS revealed that major anthocyanins belonged to cyanidin or peonidin-3-sophoroside-5-glucoside with their mono- or diacylated derivatives “caffeic, *p*-coumaric, ferulic, *p*-hydroxy-benzoic acids”. Diacylated anthocyanins with *p*-coumaric acid and caffeic acids exhibited improved biological activities, viz., antioxidant and anticarcinogenic agents compared to the anthocyanins that were diacylated with ferulic and hydroxybenzoic acids [38]. In another investigation on purple flesh sweet potatoes, the profiling of anthocyanins using LC-MS and NMR revealed its enrichment with acylated peonidin and cyanidin. The acylation with phenolic acids “caffeic and *p*-hydroxy benzoic acids” and multiple glycosidation processes showed better stability of red and blue colours not only at both acidic and basic pH but also at high temperatures, oxygen, and lighting conditions compared to the parent molecule peonidin-3-*O*-glucoside [39,40].

In a study that tested the cancer chemo-preventive potentials of polyphenolic compounds isolated from green tea in the structure–activity relationship, the results revealed that the esterification of catechin and epicatechin with gallic acid to produce catechin gallate (CG) and epicatechin gallate (ECG) improved their antiproliferative effects, while EGCG exerted the most potent antiproliferative effects on HCT-116 cells [41].

Although natural sulphated flavonoids are uncommon derivatives of secondary metabolites, they are exclusively found in specific plant species in monocotyledons and eudicotelydons that are usually enriched in plant growth in aquatic areas. The first documented sulphated flavonoid was isorhamentin-3-*O*-sulphate in *Polygonum hydropipper*. Their functions in plants are not fully elucidated, although they are likely to have impacts on the colour stabilization of anthocyanins by the formation of stable complexes as well as regulating the plant’s growth via auxins transport [42]. Chemically, sulphated flavonoids are monoesters or multiple sulphate esters of flavones and flavonols, and opposed to other acylated flavonoids, they are more polar, which may be the reason for their instability during isolation or purification. The sulphate moieties can be directly attached to flavonoid aglycone or to the sugar residue of the glycoside. For a review of sulphated flavonoids from different plant species [42], the sulphate group rendered the entire flavonoid molecule to be polar, with negative charges facilitating their water solubility, and it was observed to have more anticoagulant actions than the non-sulphated form, as in case of persicarin [43]. Likewise, quercetin-3’-sulphate showed improved anti-inflammatory actions through an improved inhibition of the COX-2 enzyme and improved antioxidant activities than the natural α-tocopherol. Sulphated flavonoids isolated from *Polygonum hydropipper* showed that the aldose reductase had a “reduction of glucose to sorbitol” inhibitory action.

## 5. Stability and Light Resistance of Acylated Flavonoids

The stability of acylated anthocyanins depends on the type of acylation. In a previous study, acylated anthocyanins of red cabbage were proved to be more stable than non-acylated ones. The type of hydroxycinnamic acid influenced the stability towards pancreatic digestion. Identified anthocyanins in red cabbage included mostly cyanidin glucosides mono- or diacylated with *p*-coumaric, ferulic, sinapic, and caffeic acids and accounted for red color stabilities in red cabbage compared to other natural resources of anthocyanins, such as roselle [44]. Acylation with sinapic acid residues reduced the stability of anthocyanins compared to other acids. Although anthocyanins are stable at the acidic pH of the stomach, they are unstable at higher pH levels of pancreatic and intestinal digestion and in conditions comprising elevated temperatures and oxygen presence [45].

A purple sweet potato was tested for its heat stability. The purple sweet potato is enriched with acylated anthocyanins including cyanidin 3-caffeoyl-*p*-hydroxybenzoyl sophoroside-5-glucoside, peonidin 3-caffeoyl sophoroside-5-glucoside, and cyanidin 3-(6’’-caffeoyl-6’’-feruloylsophoroside)-5-glucoside as major forms. Monoacylated anthocyanins showed best results in heat stability compared to non-acylated and di-acylated forms, while cyanidin 3-*p*-hydroxybenzoyl sophoroside-5-glucoside showed the best heat stability results among all acylated anthocyanins [46].

To assess the influence of the acylation of flavonoids with aromatic acids on their light resistance ability, a trial focused on the preparation of acylated flavonoids, such as isoquercetin (quercetin-3-O-*β*-glucoside) and crysanthemin (cyaniding 3-*O*-*β*-glucoside), by esterification with vinyl cinnamate- or vinyl *p*-coumarate-catalysing lipase enzymes was performed. Acylated flavonoids were tested versus their non-acylated forms for their light resistance: A “decrease in maximum absorbance under white fluorescence illumination” was used as the screening tool. *p*-coumaroyl derivatives followed by cinnamate ones were more photostable than their non-acylated glucosides as well as their aglycones “querectin and cyanidin”. The improved stability was due to the hydrophobic interaction of the polyphenolic skeleton with the aromatic acid part [47]. Another study was performed on cyanidin glycosides synthetically acylated with various acyl groups, viz., cinnamic, ferulic, sinapic, and dihdroferulic acids using lipase enzymes aimed at improving its thermal stability. The thermostability of cyanidin-3-glucoside and its corresponding purified esters was tested in a water bath at different temperatures: At 40, 60, and 85 °C, the first-order reaction rate constants (k) and half-time (t_1/2_) values were calculated. The synthetic acylated cyanidins with dihydroferuoyl and dihydrosinapoyl residues recorded improved thermal stabilities, with the most prominent effect at 85 °C possessing (k) and (t_1/2_) as 0.1391, 5.0 and 0.0728, 9.5, respectively, compared to cyanidin-3-glucoside [48].

## 6. Pharmacokinetics of Acylated Flavonoids

A study on weaning pigs fed on Marionberry rich in anthocyanins was conducted to assess their metabolism and to indicate their metabolites in urine and blood. The Marionberry extract was found to be enriched with anthocyanins, mainly including cyanidin-3-glucoside, cyanidin-3-rutinoside, and pelargonidin-3-glucoside. Metabolites of non-acylated anthocyanins, mainly of glucuronidated and methylated forms, were metabolized mainly in the liver and kidneys. The type of sugar and aglycone influenced their absorption as well as their metabolism. The acylated cyanidin was detected in urine with no metabolites; it was associated with low urinary recovery and was not extensively metabolized [49]. In another study on the absorption and metabolism of the red orange anthocyanins fed to rats for 12 days, the results revealed that cyanidin-3-glucoside and its acylated cyanidin-3 (6’’-malonyl) glucoside showed more absorptions in the stomach versus the intestine. Both cyanidin glucoside and its malonyl derivative were detected in the urine as well as their methylated metabolites, showing that both acylated and non-acylated cyanidin glucosides have the same absorption and metabolism pathways [50].

The investigation of black carrot juice enriched with both acylated (75%) and non-acylated anthocyanins was performed, where the anthocyanins of cyanidin type were found to be acylated with *p*-coumaric, ferulic, and sinapic acids. Although carrot juice was enriched mainly with acylated cyanidins, their low bioavailability was still observed rather than the non-acylated ones. Plasma levels of acylated anthocyanins were four times more than non-acylated ones due to their lower absorption levels [51]. In another study on red cabbage, the findings were in agreement with [49,51], showing that the bioavailability of acylated anthocyanins was found to be less than that of non-acylated anthocyanins. Peak plasma concentrations of non-acylated anthocyanins were four-fold higher than that for acylated anthocyanins. In this study, the intake of red cabbage at different doses by 12 volunteers was assessed to determine the anthocyanin’s bioavailability by monitoring anthocyanin metabolites in urine using HPLC-MS. Urine anthocyanins were detected as intact anthocyanins, as well as methylated and glucuronidated metabolites. The results showed that both singly and doubly acylated anthocyanins have the same recovery rate. However, for acylated anthocyanins possessing cyanidin-3-diglucoside-5-glucoside structures, there was a significant finding, and the absence of a diacylated form in urine was observed. This may be attributed to the presence of sinapoyl moieties, which inhibited its absorption. Sinapic acids rendered the compound more hydrophobic than acylated by other acyl groups, which might have negatively affected the sinapoyl anthocyanin’s absorption and bioavailability [52].

The next subsections shall describe, in detail, reports on the different chief biological effects of acylated flavonoids in relation to their main effects. Effects described in detail include hepatoprotective, anti-diabetic enzyme inhibitors (Acetyl, butyryl-choline esterase natural inhibitors, α-Amylase and α-glucosidase enzyme natural inhibitors, aldose reductase natural inhibitors, xanthine oxidase natural inhibitors, sarco/endoplasmic reticulum Ca^+2^-ATPase pump (SERCA1) natural inhibitors, human immunodeficiency virus 1 (HIV-1) integrase enzyme natural inhibitors, 5-α-reductase enzyme inhibitors, and tyrosinase inhibitors), cardioprotective activity, vasorelaxant and haematological activity, and miscellaneous activities (antifungal activity, wound healing activity, and anxiolytic activity).

## 7. Biological Effects of Natural Acylated Flavonoids

### 7.1. Hepatoprotective Action

**Figure 2:**
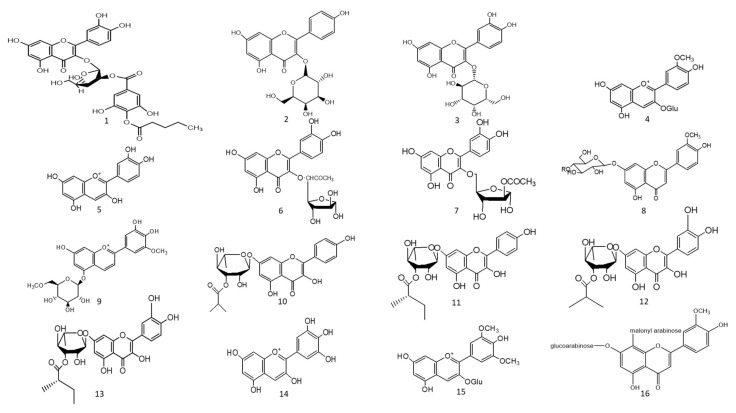

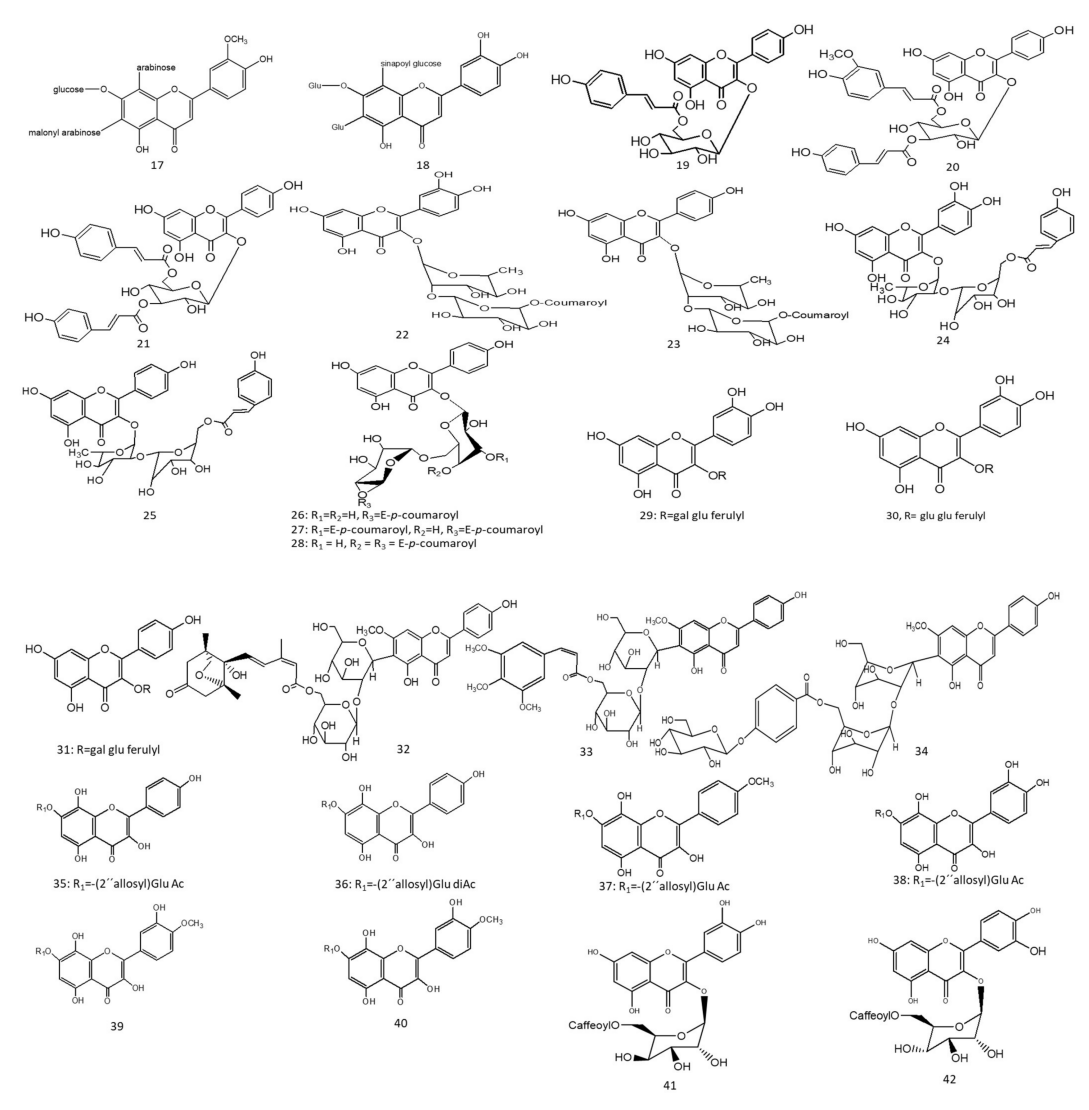

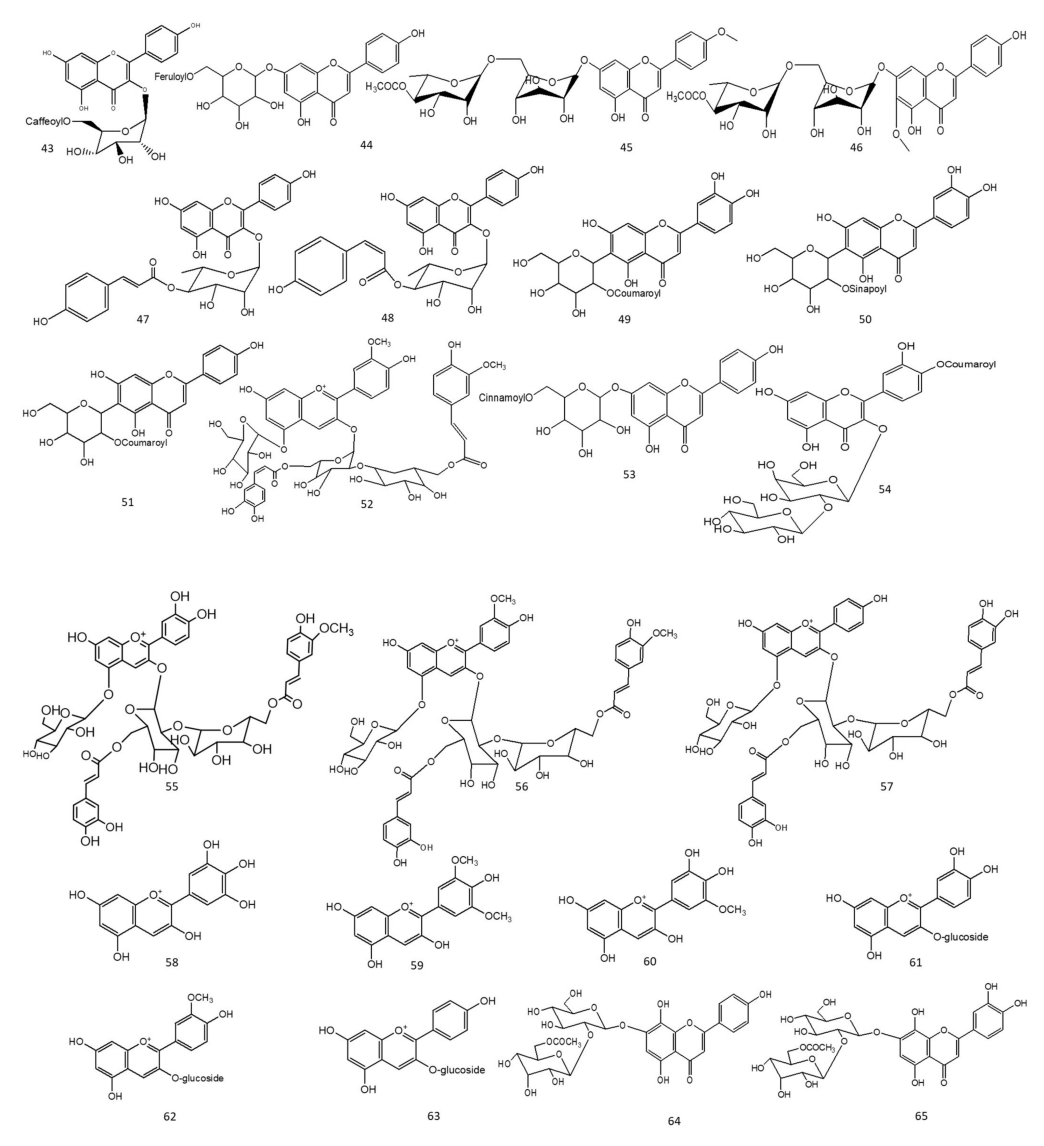

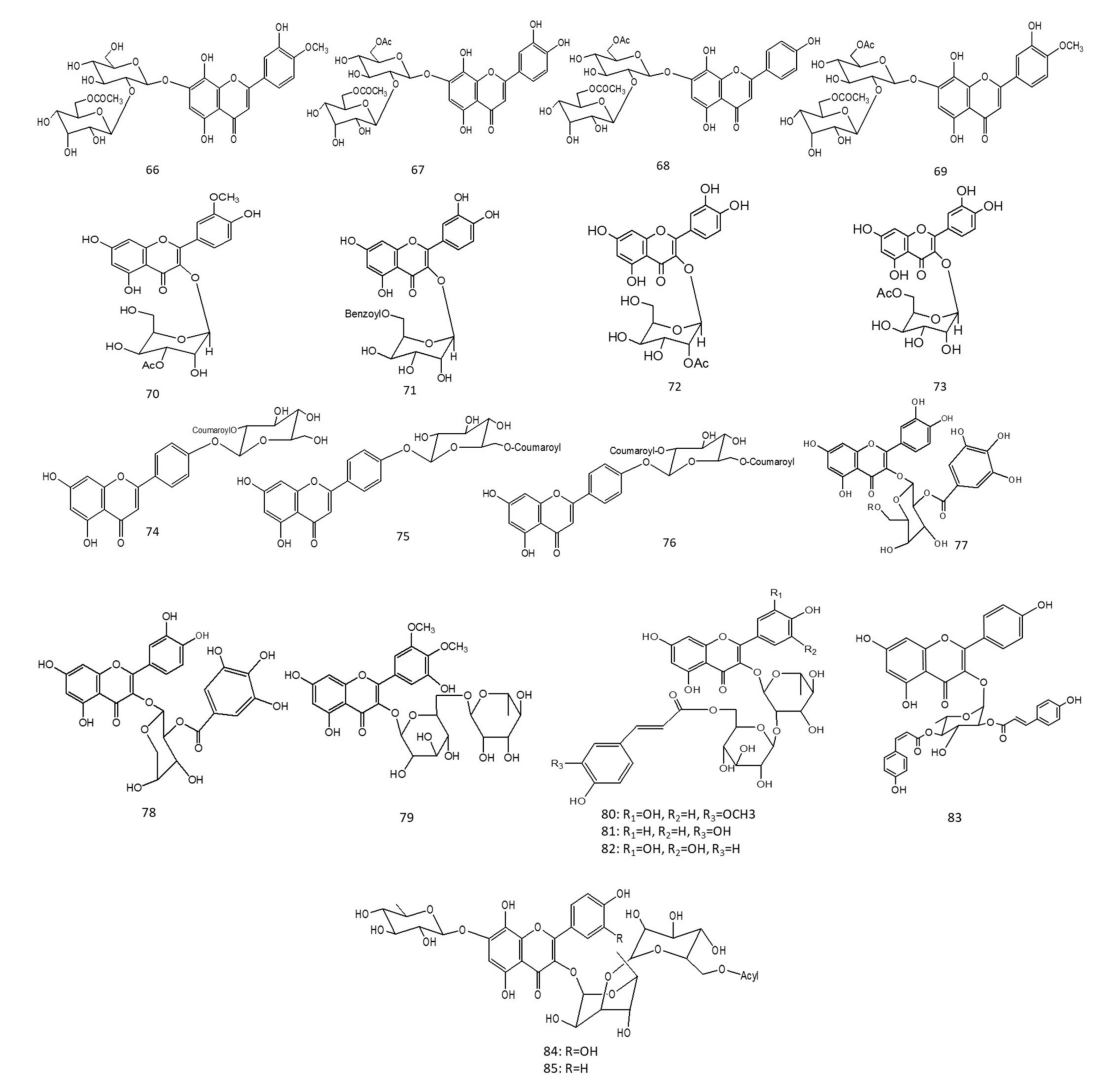
Chemical structures (1–85) of acylated flavonoids.

**Figure 3:**
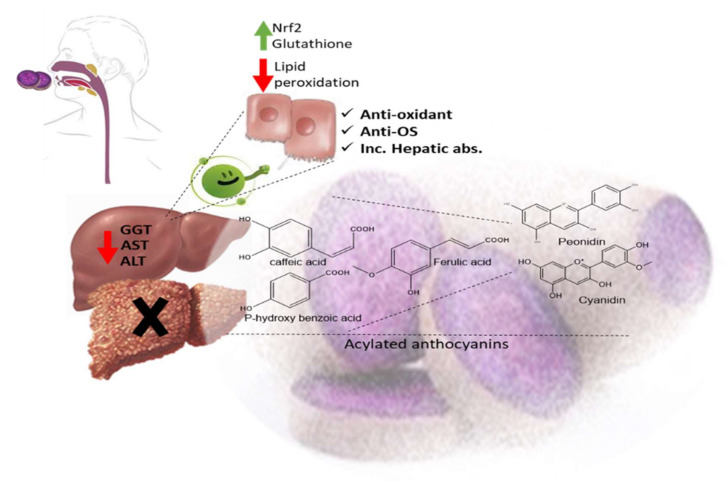
Acylated anthocyanins’ impact on hepatocytes in sweet purple potato.

**Figure 4:**
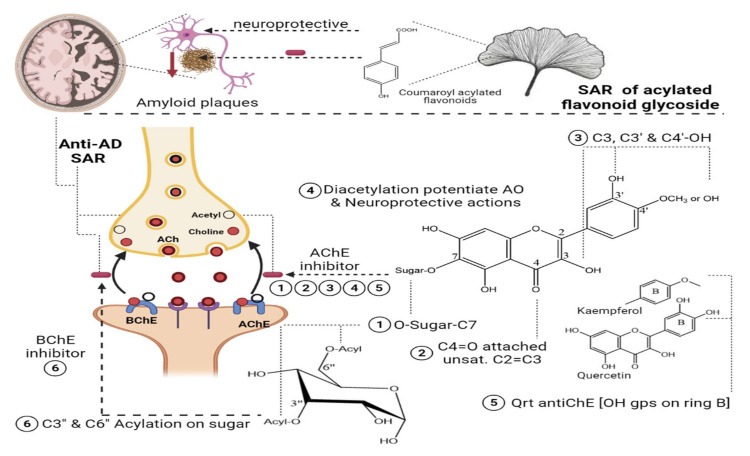
SAR of acylated flavonoid glycosides on Anti-cholinesterase with impact on AD.

**Figure 5:**
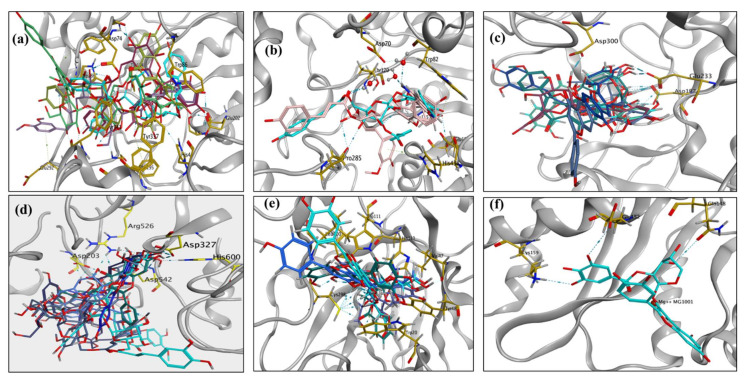
(**a**): 3D superimposition of compounds **19**, **24**, **25**, **32**, and **33** bound to acetylcholinesterase; (**b**): 3D superimposition of compounds **20** and **21** bound to butyrylcholinesterase; (**c**): 3D Superimposition of compounds **41**–**43**, **45**, and **46** bound to α-amylase; (**d**): 3D superimposition of compounds **47**, **48**, and **54**–**57** bound to α-glucosidase; (**e**): 3D superimposition of compounds **64**, **65**, **70**, and **71** bound to aldose reductase; (**f**): 3D docking pose of compound **78** bound to HIV-1 Integrase.

The ethyl acetate fraction of *Jussiaea repens’* “Primrose willow” aerial parts was found to be enriched in flavonoid aglycones “quercetin and kaempferol”, glycosides and acylated flavonoid glycosides ca. avicularin 2’’-(4’’’-*O*-*n*-pentanoyl)-gallate (Figure 2-(1)), trifolin 2’’-*O*-gallate (Figure 2-(2)), and hyperin 2’’-*O*-gallate (Figure 2-(3)). The ethyl acetate fraction exerted potential antioxidant, anti-inflammatory, and hepatoprotective effects. Elevated levels of malondialdehyde “MDA” and glutathione “GSH” of liver homogenates were reduced by 50% and 40% at 100 mg/kg body-weight doses, respectively, in comparison to the silymarin standard. The effect was correlated with high flavonoids levels, mainly in acylated galloylated forms. Acylation with gallic acid and with the number of gallic acid moieties led to improved anti-inflammatory and hepatoprotective actions [53]. In Japan, a human trial was conducted on male volunteers with borderline hepatitis upon the oral intake of the purple sweet potato beverage “*Ipomea batatas*”. The serum hepatic markers, viz., gamma glutamyl transferase “GGT”, aspartate amino transferase “AST”, and alanine transferase “ALT” decreased upon the administration of sweet potato beverages. The beverage showed enrichments in anthocyanins, mainly involving cyanidin and peonidin glycosides acylated with caffeic acid, *p*-hydroxy benzoic acid, and ferulic acid residues. The acylated anthocyanins showed potential free radical scavenging effects and improved absorption, which protected the liver from oxidative stress induced by hepatitis [54]. The high content of acylated anthocyanins in Chinese purple sweet potato “*Ipomea batatas*” belonged to peonidin (Figure 2-(4)) and cyanidin (Figure 2-(5)) acylated with phenolic acids, *viz*., caffeic, ferulic, and *p*-hydroxy benzoic acids. Sweet potato extracts exhibited cellular antioxidant activities via Nrf2 and glutathione activation and lowered the cellular lipid peroxidation of Huh7 and HepG2 hepatocytes cell lines [55], as illustrated in Figure 3.

Two acylated flavonoids isolated from *Rodgersia podophylla* A Gray (Saxifragacea) aerial parts, identified as quercetin 3-*O*-*α*-L-(5’’-*O*-acetyl)-arabinofuranoside (Figure 2-(6)), demonstrated 50.1 % hepatoporotective effects at a concentration 100 µM, and quercetin 3-*O*-*α*-L-(3’’-*O*-acetyl)-arabinofuranoside (Figure 2-(7)) exerted a 45.7 % hepatoprotective activity at a of concentration 50 µM upon testing on cultured rat hepatocytes injured by hydrogen peroxide compared to the silibinin standard [56].

### 7.2. Anti-Diabetic Action

The total alcoholic extract of *Cyperus laevigatus* aerial parts showed an effective antidiabetic action after streptozotocin (STZ) induced diabetes in rats. The antidiabetic action was proved through the inhibition of abnormal pancreatic histological changes observed in diabetic rats. *Cyperus laevigatus’* total alcoholic extract regenerated pancreatic *β*-cells responsible for insulin production. The antidiabetic markers tested for diabetic rats treated with *C. laevigatus* extracts showed inhibitions in serum glucose and glucagon levels as well as increased insulin secretions. The antidiabetic action was attributed to the enrichment of the extract with flavonoids, along with a novel acylated flavonoid diglucoside from the aerial parts named chrysoeriol 7-*O*-*β*-(6’”-*O*-acetyl-*β*-D-glucosyl)-(1→4) glucoside (Figure 2-(8)) [57]. Another study utilized the same STZ-induced diabetes model in rats to test for the antidiabetic action of the purple sweet potato extract at a dose of 165 mg/kg body weight. The antidiabetic activity of the extract was correlated with its high content of several acylated anthocyanins glycosides “up to 75 % of total anthocyanins”, mainly petunidin-3-*p*-coumaroyl-rutinosyl-5-glucoside (Figure 2-(9)). The total extract of purple sweet potato “*Ipomoea batatas*” showed decreased levels of serum glucose, glycated haemoglobin, as well as improved sugar tolerance [58]. In another human study, 17 volunteers consumed one meal enriched in mashed yellow potato diets with and without purple sweet potato “*Ipomoea batatas* (Convolvulaceae)”, which was later observed to be rich in acylated anthocyanins. The group of volunteers to consume the purple sweet potato showed postprandial lower levels of blood glucose and insulin levels, likely attributed to the high content of acylated anthocyanins represented by petunidin-*p*-coumaroyl-rutinosyl-glucoside and peonidin-*p*-coumaroyl-rutinosyl-glucoside. The proposed action mechanisms of acylated anthocyanins occur via multiple targets, including the inhibition of α-glucosidase enzymes, the inhibition of the transportation of glucose, and the modulation of carbohydrate metabolism through intracellular signalling pathways [59].

A study on the ethyl acetate fraction of *Jussiaea repens* (Onagraceae) aerial parts, antidiabetic action was recorded at a dose of 50 mg/kg animal weight through the inhibition of glucose levels in alloxan-induced diabetes in rats using glibenclamide as the standard. The antidiabetic action was in correlation to high levels of galloylated flavonoid glycosides [53]. Four new acylated flavonoids named sinocrassosides A_1_, A_2_, B_1_, and B_2_ (Figure 2-(10–13)) were isolated from the methanol extract of Chinese *Sinocrassula indica*, family Crassulaceae. The methanol extract possessed an inhibition of serum glucose levels in diabetic rats at 250 mg/kg oral dose; with the presence of these flavonoids in the extract, the antidiabetic effect was suggested to be mediated via intestinal inhibition of sugar absorptions, a potential target for the treatment of type II diabetes [60].

Anthocyanins’ chemical diversity in different types of berries and concord grapes has been examined with respect to the metabolic risks associated with obesity-related disorders, such as insulin resistance and abnormal glucose metabolism in rats fed on high-fat diets for 12 weeks. The discrepancy in biological activities was correlated with anthocyanins’ chemical diversity, including delphinidin (Figure 2-(14))/malvidin (Figure 2-(15)) versus cyanidin types. Results revealed that berry extracts rich in delphinidin and malvidin types were more effective in improving metabolic risk factors rather than cyanidin-type anthocyanins. Concord grapes were found to be enriched with acylated anthocyanins up to 54% of total anthocyanins belonging to malvidin, delphinidin, and petunidin types. The complex glycosylation and acetylation of anthocyanins reduced their bioactivity, although with increased stability in the GIT, as the disruption of gut microbiome with antibiotics led to four- to ten-fold increases in diglycosylated and acylated anthocyanins excretes. Additionally, the conversion of hydroxylated anthocyanins to the methylated analogues as malvidin was found to be crucial for their tissue uptake and ultimate biological activity [61].

### 7.3. Natural Enzyme Inhibitors

Acylated flavonoids enriched with herbs exert an inhibitory effect on key enzymes involved in several metabolic processes inside the human body. In diabetes mellitus, α-amylase and α-glucosidase inhibitors can be effective targets for controlling diabetes by delaying digestion and, therefore, intestinal absorption. Angiotensin converts enzyme inhibitors for controlling hypertension, while inhibitors of acetyl and butyryl choline esterases are for preventing symptoms of Alzheimer’s disease [62,63].

#### 7.3.1. Acetyl and Butyrylcholinesterase Natural Inhibitors “Neuroprotective Action”

Two species of *Lathyrus* “*digitatus and cicero*” revealed moderate inhibitory action on acetylcholinesterase enzymes, with a higher inhibition effect on butyrylcholinesterase enzymes. The inhibitory action of butyrylesterase enzymes on the extracts was related to its rich flavonoid content, particularly caffeoyl acylated glycosides [63]. The HPLC analysis of iced tea prepared from the aerial parts of *Spergularia rubra* (L.) (Caryophyllaceae) revealed the presence of predominant apigenin derivatives (70% of total flavones), followed by chrysoeriol and luteolin *C*-type acylated glycosides (Figure 2-(16–18)). Aromatic acylated forms included, *viz.*, *p*-coumaric, ferulic, and sinapoic acids, whereas aliphatic forms included, *viz.*, malonyl acylation. Anti-acetylcholinesterase activities were correlated mainly with *C*-flavone glycosides structure, and the presence of the methoxy group on C4’ of ring B and glycosylation on C7-OH proved to be responsible for the high AChE inhibition of these compounds, as shown in Figure 4. The preparation of iced tea beverages of *Spergularia rubra* potentiated the butyrylcholinesterase inhibition. Major acylated and non-acylated glycosides of flavones were detected in during ice-tea preparation, listed in Table 1 [62]; the identification of most active forms has not been examined yet. Acylated kaempferol glycosides (Figure 2-(19–21)), stated in Table 1**,** were isolated from the mature fronds of *Stenochlaena palustris*. They showed strong inhibitory activities on acetylcholinesterase and moderate anti-butyrylcholinesterase action. Acylation on C3’’ and C6’’ of the sugar residue contributed strongly to their anti-butyrylcholinesterase action, and acylation with *p*-coumaroyl was found to be more active than that with feruloyl moiety [64], in accordance with [65,66].

In a study on *Ginkgo biloba* flavonoids, two major acylated flavonoids (Figure 2-(22) and 23) were isolated and identified using NMR spectroscopy, as mentioned in Table 1. Both glycosides showed a neuroprotective potential and delayed the progression of Alzheimer’s disease (AD) through moderate to weak Aβ fibril peptide deposition inhibition action, which is considered as a rate-limiting step in delaying AD neurodegenerative progression. The presence of acylated flavonoids in ginkgo leaves rather than triterpene lactones appeared to be a determinant for the anti-AD neurodegenerative disorder, with the acylated flavonoid being more potent than its non-acylated counterpart. Specifically, the presence of the *p*-coumaroyl group potentiated the formation of non-covalent binding with amyloid-*β* structure peptides [66], as shown in Figure 4. In another study, the chemical profiling of *Gingko biloba* extracts revealed the high content of acylated flavonols representing 4.5% of the entire extract, including quercetin cinnamoyl glycoside (Figure 2-(24)**)** and kaempferol cinnamoyl glycoside (Figure 2-(25)). The presence of acylated quercetin and kaempferol flavonoids [67] enriched in *gingko* extracts potentiated the cognitive properties. The presence of an extra methoxy group in *ginkgo* acylated flavonol represented by isorhamnetin proved to have no influence in the increment of acetylcholine and dopamine levels upon using *ginkgo* extract “EGB761” in rats’ medial prefrontal cortex [68].

Nevertheless, such hypotheses cannot be generalized for other acylated flavonol glycosides, viz., kaempferol coumaroyl glycosides (Figure 2-(26–28)) (listed in Table 1) isolated from flowers of *Aerva javanica*. *p*-coumaroyl kaempferol glycosides were tested for inhibition against cholinesterase, butyryl esterase, and lipoxygenase enzymes showing weak activity against the aforementioned enzymes [65]. Among acylated flavonol glycosides, quercetin analogues were more active than kaempferol as neuroprotective agents (Figure 2-(31), R as Figure 2-(29,30)) in neurotoxicity glutamate assays. The presence of dihydroxy groups in ring B of flavonoid nucleus, illustrated in Figure 4, as well as acylation was found to be essential for enhanced protective action, whereas the nature of sugar residues had no influence on neuroprotective actions [69], and this is in accordance with previous reports highlighting the potential activity of quercetin versus kaempferol [65,66].

In a study aimed at assessing the anti-cholinesterase activity of *Ziziphus mauritiana* flavonoids, three new acylated spinosins (Figure 2-(32–34)), elaborated in Table 1, were identified and showed moderate inhibitory impacts on acetylcholinesterase enzymes [70]. Upon searching for non-alkaloid nucleus-controlling symptoms of (AD), the isolation of acetylated flavonoids from the ethyl acetate fraction of *Galeopsis ladanum* L. (Lamiaceae) leaf yielded six acetylated flavonoids, three of which were acetylated isoscutellarein glycosides (Figure 2-(35–37)), and the others were acetylated hypolaetin glycosides (Figure 2-(38–40)), as stated in Table 1. SAR among the isolated flavonoids identified three structural motifs that are crucial for the anti-AD activity related to their free radical scavenging property, as depicted in Figure 4. Furthermore, DPPH assays revealed potentiation of the antioxidant property in diacetylated compounds (Figure 2-(36,40)), exhibiting IC_50max_ of 5.6 µg/mL and 7.4 µg/mL, respectively, compared to monoacetylated glycosides (35, 39), showing IC_50max_ of 6.4 µg/mL and 10.6 µg/mL, respectively, whereas an opposite pattern was observed for anti-cholinesterase activity. An acetylcholinesterase inhibition assay was performed by TLC, where only monoacetylated isoscutellarein-4’-methyl ether-7-(2’’-allosyl)-glucoside (Figure 2-(37)) and monoacetylated hypolaetin-4’-methyl ether-7-(2’’-allosyl)-glucoside (Figure 2-(39)) possessed inhibitory activity on the choline esterase compared to galantamine as a reference. Results suggested that C4’ methylation with mono-acetylation was crucial for anticholinesterase inhibition and neuroprotective functions [71,72].

**Table 1:** Plants having neuroprotective action “Acetyl, butyrylcholinesterase natural inhibitors”.

Acylated Flavonoids	Plant Source	Biological Action	Reference
Caffeoyl acylated glycosides	*Lathyrus digitatus Lathyrus cicero*	High inhibitory effect on butyrylcholinestearse enzyme.	[63]
7-*O*-glucosyl-6-arabinosyl-8-C-(6’’’-malonyl)-arabinosyl chrysoeriol (16), 7-*O*-glucosyl-6-C-(2’’-malonyl)-arabinosyl-8-C-arabinosyl chrysoeriol (17), 7-*O*-glucosyl-6-Cglucosyl-8-C-(2’’’-sinapoyl)glucosyl luteolin (18)	*Spergularia rubra*	Inhibitory effect on butyrylcholinestearse enzymes	[62]
Kaempferol 3-*O*-(6’’-O-E-*p*-coumaroyl)-β-glucopyranoside (19) Kaempferol 3-*O*-(3’’-*O*-E-*p*-coumaroyl)-(6’’-*O*-E-feruloyl)-*β* –glucopyranoside (20) ,Kaempferol 3-*O*-(3’’,6’’-di-*O*-E-*p*-coumaroyl)-*β* –glucopyranoside (21)	*Stenochlaena palustris*	-Strong inhibitory activity on acetylcholinesterase -Moderate anti-butyrylcholinesterase action	[64]
Quercetin-3-*O*-α-(6’’’-*p*-coumaroyl-glucosyl-*β*-1,2-rhamnoside),(22) Kaempferol 3-*O*-*α*-(6’’’-*p*-coumaroyl-glucosyl-β-1,2-rhamnoside)(23)	*Ginkgo biloba*	Neuroprotective action and anti-AD	[66]
Quercetin-3-*O*-(2’’-*O*-(6’’’-*O*-(p-hydroxy-trans-cinnamoyl)-*β*-D-glucosyl)-*α*-L-rhamnoside) (24) Kaempferol-3-*O*-(2’’-*O*-(6’’’-*O*-(*p*-hydroxy-trans-cinnamoyl)-*β*-D-glucosyl)-*α*-L-rhamnoside (25)	*Ginkgo biloba*	Increased dopamine and acetyl choline neurotransmitters alleviating cognitive properties	[67]
Kaempferol-3-*O*-*β*-D-[4’’’-*E*-*p*-coumaroyl-*α*-L-rhamnosyl(1 → 6)]-galactoside (26), Kaempferol-3-*O*-β-D-[4’’’-*E*-*p*-coumaroyl-*α*-L-rhamnosyl (1 → 6)]-(3’’-*E*-*p*-coumaroyl)-galactoside (27), Kaempferol-3-*O*-*β*-D-[4’’’-*E*-*p*-coumaroyl-*α*-L-rhamnosyl-(1 → 6)]-(4’’-*E*-*p*-coumaroyl)-galactoside (28)	*Aerva javanica*	Weak inhibitory activity against cholinesterase, butyryl esterase, and lipoxygenase enzymes	[65]
Quercetin-3-*O*-[2-*O*-(6-*O*-*E*-feruloyl)-*β*-D-glucopyranosyl]-*β*-D-galactopyranoside (29), Quercetin-3-*O*-[2-O-(6-*O*-*E*-feruloyl)-*β*-D-glucopyranosyl]-*β*-D-glucopyranoside (30), Kaempferol 3-*O*-[2-*O*-(6-*O*-*E*-feruloyl)-*β*-D-glucopyranosyl]-*β*-D-galactopyranoside (31)	*Hedyotis diffusa* Willd	Neuroprotective action on rat cortical cells injured by glutamate	[69]
6’’’-(–)-phaseoylspinosin (32) 6’’’-(3’’’’, 4’’’’, 5’’’’-trimethoxyl)-(*E*)-cinnamoyl spinosin (33) 6’’’-(4’’’’-*O*-*β*-D-glucopyranosyl)-benzoyl-spinosin, (34)	*Ziziphus mauritiana*	Moderate inhibitory impact on acetylcholinesterase	[70]
Isoscutellarein-7-(2’’-allosyl)-glucoside monoacetylated (35), Isoscutellarein-7-(2’’-allosyl)-glucoside diacetylated (36), Isoscutellarein-4’-methyl ether-7-(2’’-allosyl)-glucoside monoacetylated (37), Hypolaetin-7-(2’’-allosyl)-glucoside monoacetylated (38), Hypolaetin-4’-methyl ether-7-(2’’-allosyl)-glucoside monoacetylated (39), Hypolaetin-4’-methyl ether-7-(2’’-allosyl)-glucoside diacetylated (40),	*Galeopsis ladanum*	Antioxidant, anticholinesterase activity, and neuroprotective	[71]

#### 7.3.2. α-Amylase and α-Glucosidase Enzyme Natural Inhibitors

A study on two species of *Lathyrus*: *L. digitatus and L. cicero* revealed moderate inhibitions on α-amylase and stronger actions on α-glucosidase enzymes. Metabolite profiling revealed enrichments in extracts with flavonoids mainly acylated with quercetin and kaempferol glycosides [63]. In another study conducted on the ethyl acetate fraction of *Spiraea salicifolia* L. (Rosaceae family), leaf extracts native to Siberia revealed an abundance of caffeoyl-acylated flavonoids (Figure 2-(41–43)), as listed in Table 2. Caffeoyl-esterified flavonoids illustrated a positive impact on α amylase enzyme inhibition contributing to the promising impact of Siberian extracts as natural and effective anti-diabetic agents [73]. The prepared ice-tea beverage of *Spergularia rubra* aerial parts was found to be enriched in *C*-glycosylated flavones, including both “acylated and non-acylated” aromatic and aliphatic residues. The authors correlated the structural activity to the enzyme inhibition potential through the unsaturation of C2 and C3, as well as the hydroxyl groups present on C5 and C4’ of the acylated flavones structure. Acylation improved the stability, the solubility of the *C*-type flavone glycosides, and the ultimate bioactivity [62]. On the other hand, a study on baked Chinese green tea named Lu’an GuaPian (*Camellia sinensis* L.O. Kuntze) examined its potential inhibition of α-glucosidase and α-amylase enzymes. The baked green-tea chemical analysis demonstrated a diversity of acylated and non-acylated kaempferol and quercetin glycosides. The SAR of green tea antidiabetic activity showed that the presence of the C3’ hydroxyl group on ring C of flavonoid skeletons is crucial for its activity. The aglycone itself was more active than the glycoside, and this is most likely due to the steric hindrance of the molecule upon binding with the enzyme. The acylation phenomenon lowered the inhibitory action on the enzymes, suggesting that acylation is not critical for enzyme inhibition and, thus, antidiabetic activities [74]. Whether such patterns exist in other flavonol glycosides has not been confirmed. In a study on sweet potato extracts enriched in acylated peonidin glycosides, it markedly decreased the activity of carbohydrates enzymes, viz., α amylase and α glucosidase, to influence the carbohydrate’s metabolism and blood glucose levels [55]. An intestinal rat α-glucosidase inhibition assay was conducted on red vinegar prepared from the fermentation of purple sweet potato; the fermented vinegar contained several acylated sophoroses, acylated and non-acylated cyanidin, and peonidin glycosides. Acylated residues in both sophoroses and anthocyanins were identified to be aromatic residues, i.e., *p*-hydroxybenzoic, ferulic, and caffeic acids, and were found to improve α-glucosidase enzyme inhibition effects compared to non-acylated ones. Results revealed that newly identified(6-O-(*E*)-Caffeoyl-(2-O-(6-O-(*E*)-caffeoyl)-*β*-D-glucopyranosyl)-α-D-glucopyranose and 6-O-(*E*)-Caffeoyl-(2-O-(6-O-(*E*)-feruloyl)-*β*-D-glucopyranosyl)-α-D-glucopyranose revealed a potential for retarding the action of intestinal maltase with an IC_50_ value of 214 and 289 μM, respectively, thus proving that diacylated sophoroses preferably inhibited maltase rather than sucrase with an IC_50_ value of less than 300 µM [75]. These results opposed the findings documented in (Hua, Zhou et al., 2018), reporting that the acylation of kaempferol and quercetin glycosides obtained from green tea lowered enzymatic inhibition activities and, thus, lowering antidiabetic effects. Another study isolated caffeoylsophorose (Figure 2-(44)), as mentioned in Table 2, from red vinegar through fermentation with roots of purple sweet potatoes as a potent maltase inhibitor. In vitro and in vivo studies were conducted to understand the potential inhibitory activity of caffeoylsophoroside derived from diacetylated anthocyanins. With respect to the results revealed in the in vitro study, caffeoylsophoroside recorded an effective inhibition of *α*-glucosidase (maltase and sucrase) rather than *α* amylase recording IC_50_ ca. 699 ± 17.1, 874 ± 39.0, and 25,200 ± 11,500.0 µM. In the in vivo model, the acylated sugar lowered the blood glucose level, with caffeoyl sugar found to suppress glucose absorptions rather than increasing insulin sensitivity or even glucose transport inhibitions upon comparisons to a control group that only had a solution of the substrate (maltose, glucose, or sucrose) administered without the caffeoylsophorose compound. SAR confirmed the crucial role of the acyl residue “caffeic acid” within the sugar structure in addition to the hydroxy groups and the unsaturated alkyl chain in caffeic acid. The authors suggested the use of caffeoyl sophoroside as a supplement with oral conventional hypoglycaemic drugs for controlling diabetes [76], which has yet been assessed at clinical levels.

A study on Iranian *Zhumeria majdae* (F. Lamiaceae) was conducted, which is commonly used in the treatment of stomach pain as well as dysmenorrhea. Two acylated flavonoid glycosides (Figure 2-(45,46)) isolated from the butanol fraction, Table 2, were identified. The activity of *α*-amylase enzyme was dose-dependently suppressed by the butanol extract. The butanol fraction exhibited the highest inhibition at 30 mg/mL towards the enzyme (77.9 ± 2.1%), while acarbose inhibited the enzyme at 20 mg/mL by 73.9 ± 1.9%. The IC_50_ for the butanol fraction and methanol extracts was calculated at 24.5 ± 2.1 and 22.0 ± 2.7 mg/mL, respectively, compared to acarbose (6.6 ± 3.1 mg/mL) as the positive standard. This potential in vitro *α*-amylase inhibition could be an effective potential nutraceutical for controlling diabetes [77]. A study in Taiwan led to the isolation of two acylated flavonols, identified as kaempferol-coumaric acid esters (Figure 2-(47,48)), as shown in Table 2, from *Machilus philippinense* leavf extracts, and they possess potential α-glucosidase inhibition. The study proved the critical role of coumaroyl esters in such effects, and acylation with di-*p*-coumaric acid in both *trans* and *cis* configurations was more active than di-*p*-coumaric acid in only trans configuration [78]. A bio-guided fractionation study aimed for the isolation of active components of *Tinospora crispa* Miers (Menispermaceae) traditionally used for the treatment of diabetes through the activation of insulin signalling. The fractionation led to the isolation of several acylated flavonoids (Figure 2-(49–53)), listed in Table 2, and they were identified by spectroscopic techniques. The acylated flavonoids recorded an effective IC_50_ of 4.3 µM mainly for isovitexin-*p*-coumarate (Figure 2-(51)); the presence of hydroxyl group on C3’ “isoorientin coumarate (Figure 2-(49))” lowered the hypoglycaemic activity compared to its C3’ dehydroxylated one, “isovitexin coumarate”, whereas the stereochemistry did not alter the inhibition activity of the α-glucosidase enzyme [79].

A novel acylated quercetin glycoside was isolated from Chinese *Panax ginseng* as floralpanasenoside A, a benzoyl quercetin glycoside (Figure 2-(54)), as shown in Table 2. The glycoside recorded potential inhibitory actions against the α-glucosidase enzyme (IC_50_ =62.4 µM) with IC_50_ values lower than the positive control acarbose (385.2 µM). The in silico docking model revealed that panasenoside A bounded with numerous amino acids on the pocket active sites on α-glucosidase via hydrogen bonds [80]. A phytochemical investigation of the red flowers of morning glory “*Pharbitis nil* cv.” and storage roots of purple sweet potato “*Ipomoea batatas* cv. Ayamurasaki” led to the identification of active acylated anthocyanins (Figure 2-(55–57)) with hypoglycaemic activities, as mentioned in Table 2. Results revealed that the acylation of anthocyanins is crucial for α-glucosidase enzyme inhibition, emphasizing acylation with caffeoyl or feruyl moieties for maltase inhibition [81]. In another investigation, three cultivars of blueberries and their impact on in vitro α-glucosidase enzyme inhibition were studied; they showed promising inhibitory activities, including the Northland cvs. enriched with anthocyanins that are either acylated or non-acylated. The acylated anthocyanins were identified as C6’’-acetylated C3-glycosides of delphinidin (Figure 2-(58)), malvinidin (Figure 2-(59)) and petunidin (Figure 2-(60)) types. Although acylated anthocyanins appeared more determinant than non-acylated glycosides in α-glucosidase inhibition, emphasizing the crucial role of acylated residues of “caffeic or ferulic acid”, the presence of free phenolic acids in the extracts showed stronger α glucosidase inhibition activity [82].

A study aimed to correlate the phenolic composition of 20 genotypes of purple maize (*Zea Mays* L.) as hypoglycaemic agents via the inhibition of α glucosidase enzymes. LC-MS profiling revealed the presence of major acylated anthocyanin 3-O-glucosides belonging to malonyl esters at C6’ of cyaniding (Figure 2-(61)), pelargonidin (Figure 2-(62)), and peonidin (Figure 2-(63)), as shown in Table 2. The hypoglycaemic action of *Zea mays* extracts correlated with the modulation of diabetes-related enzymes with “α-glucosidase inhibition” led to further improvements in insulin sensitivity in adipocytes [83], suggesting a synergistic action.

**Table 2:** α-Amylase and α-glucosidase enzyme natural inhibitors.

Acylated Flavonoids	Plant Source	Biological Action	Reference
6’’-*O*-caffeoyl-hyperoside (41), 6’’-*O*-caffeoyl isoquercitrin (42), 6’’-*O*-caffeoyl-astragalin (43)	*Spiraea salicifolia*	Inhibitory effects on α-amylase, natural antidiabetic agent	[73]
6-*O*-(*E*)-caffeoyl-2-*O*-*β*-D-glucopyranosyl-*β*-D-glucopyranoside (6-O-caffeoylsophorose) (44)	Red vinegar fermented with roots of purple-sweet potato enriched with peonidin-3-O-(2-O-(6-O-E-feruloyl-β-D-glucopyranosyl)-6-O-E-caffeoyl-β-D-glucopyranoside)-5-O-β-D-glucopyranoside)	Inhibitory effects on maltase and sucrase enzymes; no effect on α amylase; the acylated penoidin possessed an effective inhibitory action on α amylase	[76]
Linarin (45) Hispidulin-7-*O*-(4-*O*-acetyl-rutinoside)(46)	*Zhumeria majdae*	Treatment of stomach pain and dysmenorrhea Potential inhibitory action on α amylase	[77]
kaempferol-3-*O*-*α*-L-rhamnopyranoside 3’’,4’’-di-*E*-*p*-coumaric acid ester (47) Kaempferol-3-*O*-*α*-L-rhamnopyranoside-3’’-*E*,4’’-*Z*-di-*p*-coumaric acid ester (48)	*Machilus philippinense* Merr,	Potent α-glucosidase inhibition	[78]
Isoorientin 2’’-(*E*)-*p*-coumarate (49), Isoorientin 2”-*O*-(*E*)-sinapate (50), Isovitexin 2”-(*E*)-*p*-coumarate (51), Cosmosiin-6’’-(*E*)-ferulate (52), Cosmosiin 6’’-(*E*)-cinnamate (53)	*Tinospora crispa* Miers	Antidiabetic through activation of insulin signaling	[79]
Panasenoside A (54) (quercetin-4’-*p*-hydroxybenzoyl-3-*O*-(2’’-*β*-D-glucopyranosyl)-*β*-D-galactopyranoside)	Chinese *Panax ginseng*	Inhibitory activity against α-glucosidase	[80]
Cyanidin (55) Peonidine-3-*O*-(2-*O*-(6-*O*-*E*-Feruyl-*β*-D-glucopyranosyl)-6-*O*-*E*-Caffeoyl-*β*-D-glucopyranoside)-5-*O*-*β*-D-glucopyranoside (56) Pelargonidin-3-*O*-(2-*O*-(6-*O*-*E*-*O*-Caffeoyl-*β*-D-glucopyranosyl)-6-*O*-*E*-Caffeoyl-*β*-D-glucopyranoside)-5-*O*-*β*-D-glucopyranoside (57)	*Ipomoea batatas cv.* Ayamurasaki *Pharbitis nil cv.*	Hypoglycemic activity by inhibition of α-glucosidase	[81]
C6’’-acetylated C3-glycosides of delphinidine (58), malvinidin (59) and petunidin (60) types	Three cultivars of blueberry	Inhibition of α-glucosidase	[82]
Cyanidin-3-*O*-glucoside (61), Pelargonidin-3-*O*-glucoside (62), Peonidin-3-glucosides (63) and their corresponding malonyl esters “C6’ acylation with malonyl residue”	*Zea Mays* L.	Hypoglycemic activity through inhibition of α-glucosidase and improved insulin sensitivity in adipocytes resistant to insulin	[83]

#### 7.3.3. Aldose Reductase Enzyme Natural Inhibitors

A study conducted on hot herbal-tea beverages of aerial *Sideritis brevibracteata* used in the Turkish folk medicine resulted in the isolation of six acetylated 8-hydroxyflavone glycosides. The six acetylated flavone glycosides were identified as isoscutellarein 7-*O*-[6’’’-*O*-acetyl-*β*-D-allopyranosyl-(1→2)]-*β*-D-glucopyranoside (Figure 2-(64)); hypolaetin 7-*O*-[6’’’-*O*-acetyl-*β*-D-allopyranosyl-(1→2)]-*β*-D-glucopyranoside (Figure 2-(65)); 3’-hydroxy-4’-*O*-methylisoscutellarein 7-*O*-[6’’’-*O*-acetyl-*β*-D-allopyranosyl-(1 → 2)]-*β*-D-glucopyranoside (Figure 2-(66)); hypolaetin 7-*O*-[6’’’-*O*-acetyl-*β*-D-allopyranosyl-(1→2)]-6’’-*O*-acetyl-*β*-D-glucopyranoside (Figure 2-(67)), isoscutellarein 7-*O*-[6’’’-*O*-acetyl-*β*-D-allopyranosyl-(1 → 2)]-6’’-*O*-acetyl-*β*-D-glucopyranoside (Figure 2-(68)); and 3’-hydroxy-4’-*O*-methylisoscutellarein-7-*O*-[6’’’-*O*-acetyl-*β*-D-allopyranosyl-(1→2)]-6’’-*O*-acetyl-*β*-D-glucopyranoside (Figure 2-(69)). IC_50_ values of Hypolaetin (0.61 µg/mL), isoscutellarein (1.16 µg/mL) and their methyl ether derivatives (1.25 µg/mL) showed almost similar inhibitory activities on human aldose reductase enzymes, except for diacetylated 3’-hydroxy-4’-O-methylisoscutellarein, which was found to be inactive with an IC_50_ value (>1000). A clear SAR among the isolated flavonoids with regards to their inhibitory potential on aldose reductase failed to be revealed [84].

Another study focused on the isolation of quercetin-acylated glycosides from flower buds of Chinese *Prunus mume,* and the identified quercetin derivatives are, viz., mumeflavonoside A (Figure 2-(70)), quercetin 3-*O*-(6’’-*O*-benzoyl)-*β*-D-galactopyranoside (Figure 2-(71)), quercetin 3-*O*-(2’’-*O*-acetyl)-*β*-D-glucopyranoside (Figure 2-(72)), and quercetin 3-*O*-(6’’-*O*-acetyl)-*β*-D-glucopyranoside (Figure 2-(73)). Acylated quercetin glycosides showed significant rat lens aldose reductase inhibition that catalysed the conversion of glucose to sorbitol, with a later accumulation that could lead to some diabetes complications, such as cataract [85].

#### 7.3.4. Xanthine Oxidase Enzyme Natural Inhibitors

A study on club moss *Palhinhaea cernua* commonly used in Chinese traditional medicine was performed by targeting its acylated apigenin glucosides and their impact on xanthine oxidase (XO) inhibition. XO enzyme plays a significant role in the breakdown of xanthines to uric acid, leading to hyperuricemia, and could be a risk factor of several diseases. The identified flavonoids were apigenin-4’-*O*-glucosides with different types of *p*-coumaroyl acylation patterns annotated as apigenin-4’-*O*-(2’’-*O*-*p*-coumaroyl)-*β*-D-glucoside (Figure 2-(74)), apigenin-4’-*O*-(6’’-*O*-*p*-coumaroyl)-*β*-D-glucoside (Figure 2-(75)), and apigenin-4’-*O*-(2’’, 6′’-di-O-*p*-coumaroyl)-*β*-D-glucoside (Figure 2-(76)). Bioassays of apigenin-acylated glycosides on XO inhibition concluded that only apigenin-4’-*O*-(2’’-*O*-*p*-coumaroyl)-*β*-D-glucoside showed potential effects with the type of acylation being crucial for its activity [86]. The XO enzyme, responsible for the release of superoxide radicals through a breakdown of xanthines to uric acid, was inhibited by the sweet potato extract “*Ipomea batatas*”, which was enriched in acylated peonidin glycosides, and acylated residues were identified as caffeic, ferulic, and *p*-hydroxy benzoic acids. The authors correlated the enzyme inhibition to the presence of hydroxy groups on C5 and C7 of ring A rather than the hydroxy groups on ring B [55].

In a study of purple sweet potato extracts enriched with highly acylated anthocyanins of cyanidin and peonidin types on alleviating hyperuricemia in rats induced by potassium oxonate, results showed that potassium oxonate significantly (*p* < 0.05) increased the protein levels of the following renal inflammatory cytokines TNF-α, TGF-*β*1, IL-6, and IL-1*β* in hyperuricemic mice. The administration of highly acylated anthocyanins from purple sweet potato (HAA-PSP) could significantly (*p* < 0.05) inhibit the expression of the protein of the previously mentioned inflammatory cytokine levels in the kidney, whether used alone or in combination with allopurinol. The concomitant administration of 25 mg/kg of HAA-PSP and 2.5 mg/kg of allopurinol resulted in the inhibition of renal biomarkers reflecting inflammation and renal damage, i.e., the inhibition of inflammatory cytokines as IL-6, TNFα, and NF-κB [87], suggestive for a synergistic effect, thus inhibiting xanthine oxidase enzymes. [88]

### 7.4. Sarco/Endoplasmic Reticulum Ca^2+^-ATPase Pump (SERCA1) Natural Inhibitors

This enzyme plays a key role in controlling muscle contractions as well as keeping calcium within normal levels in body cells. In a study assessing the influence of esterification of rutin with long chain fatty acids (C16-C22) versus the parent glycoside in inhibiting SERCA1 enzyme. Results revealed that the presence of poly hydroxyl groups in the flavonoid structure was essential for the inhibition activity, particularly on C3 and C6. Inhibition was further potentiated upon the derivatization of rutin molecules with lipophilic residues. The inhibition activity of SERCA1 also was correlated to both the chain length and unsaturation of the fatty acid molecule. Fatty acid moiety in rutin inhibited the SERCA1 activity via binding to transmembrane region as well as a loss of the thiol group, which is crucial for enzyme activities [89].

### 7.5. Human Immunodeficiency Virus 1 (HIV-1) Integrase Enzyme Natural Inhibitors

A guided fractionation of Korean *Acer okamotoanum* Nakai leaf extracts resulted in the isolation of acylated flavonoids, including quercetin 3-*O*-(2’’,6’’-*O*-digalloyl)-*β*-D galactopyranoside (Figure 2-(77)) and quercetin 3-*O*-(2’’-galloyl)-*α*-L-arabinopyranoside (Figure 2-(78)), where the two galloylated quercetin glycosides recorded effective IC_50_ at 24.2 and 18.1 µg/mL, respectively, against the (HIV-1) integrase enzyme. This enzyme is responsible for insertion of the HIV viral DNA into the DNA of host cells; inhibiting its function is a potential target in the treatment of AIDS disease [90].

### 7.6. Cardioprotective Activity

New acylated flavonoids isolated from *Gingko biloba* leaf extracts were identified as 5,7,5’-trihydroxy-3’,4’-dimethoxyflavonol-3-*O*-*α*-L-rhamnopyranosyl-(1→6)-*β*-D-glucopyranoside (Figure 2-(79)); quercetin-3-*O*-(6-(*E*)-feruloyl)-*β*-D-glucopyranosyl-(1→2)-*α*-L-rhamnopyranoside (Figure 2-(80)); kaempferol-3-*O*-(6-(*E*)-caffeoyl)-*β*-D-glucopyranosyl-(1→2)-*α*-L-rhamnopyranoside (Figure 2-(81)); and myricetin-3-*O*-(6-(*E*)-*p*-coumaroyl)-*β*-D-glucopyranosyl-(1→2)-*α*-L-rhamnopyranoside (Figure 2-(82)). The cardioprotective effect of the isolated flavonoids was tested against hydrogen peroxide-induced apoptosis in H9c2 cells, with non-acylated flavonoids being found more active than the acylated ones at 50 µM concentrations [91] and opposite to the pattern that was observed in other effects. In contrast, the semi-synthesis of the acylated isoflavone puerarin “daidzein-8-C-glucoside” with an alkyl group enhanced the cardioprotective efficacy of puerarin both in vitro and in vivo. Puerarin isoflavones were present in the roots of Chinese *Pueraria lobata*, traditionally known for its cardioprotective action. The acylation occurred on the phenolic OH groups of C4’ and/or C7 [92]. The discrepancy between [91] and [92] can be correlated to the SAR of the aglycone moiety itself regardless of acylation. Moreover, the type and position of acylated group “aromatic versus alkyl” type may also influence the cardioprotective activity.

*Laurus nobilis* L. (fam. Lauraceae), a Mediterranean plant enriched in acylated flavonoids, mostly including coumaroyl flavonoids of all kaempferol types, was studied. The cardioprotective action of the acylated glycosides was investigated via the inhibitory action of sodium, potassium adenosine tri phosphatase enzyme (Na^+^, K^+^ ATPase). The enzyme (Na^+^, K^+^ ATPase) is essential in maintaining high potassium and low sodium levels inside the cells. Inhibitory actions on such enzymes propose lines of treatment for various cardiovascular disorders. All acylated kaempferol glycosides of *Laurus nobilis* leaf extract recorded significant inhibitory actions, with kaempferol-3-*O*-*α*-L-(2’’-*E*, 4’’-*Z*-di-*p*-coumaroyl)-rhamnoside (Figure 2-(83)) being the most active with an IC_50_ of 5.0±0.1 µM compared to the Ouabain drug, which was used as a positive control. Results revealed that *p*-coumaric acid conjugated to C3’’ of rhamnose residue in the “*E* isomer” exerted better results than “*Z* isomer”; additionally, the presence of the two isomers of *p*-coumaric acid on C2’’ and C4’’ in the sugar moiety enhanced its inhibitory activity. The presence of both *p*-coumaroyl groups and kaempferol aglycone was required to exert synergetic inhibitory actions in acylated kaempferol glycoside rather than *p*-coumaroyl or kaempferol aglycone when each was tested alone [93].

### 7.7. Vasorelaxant and Haematological Activity

A Taiwan leaf pine, *Pinus morrisonicola*, was assessed for its vasorelaxant activity by blocking the voltage-operated calcium channel. LC-MS profiling identified three coumaroyl flavonoids of quercetin and kaempferol glycosides, along with a novel kaempferol 3-*O*-*α*-(6’’’-*p*-coumaroyl-glucosyl-*β*-1, 4-rhamnoside), which exerted a significant inhibition of Ca2+ fluorescence with IC_50_ values of 0.20 mM, proving a vasorelaxant effect by blocking the voltage-operated Ca^2+^ channel and inhibiting Ca^2+^ influx to the cytoplasm. Results suggested the use of pine leaves and its major components as natural anti-hypertensive agents [94]. Another study on the haemodynamics of black carrot extracts enriched with acylated anthocyanins, mostly including aromatic acylation, viz., sinapoyl, coumaroyl, and ferouyl residues, showed an increase in rat arteriolar blood flow upon single intragastric doses of the acylated enriched extract compared to non-acylated cyanidin and delphinidin glycosides. The aglycone’s nature was also found to influence its haemodynamics with delphinidin glycoside, which is more active compared to cyanidin [95]. Leaves of *Flaveria bidentis* were enriched in sulphated flavonoids; i.e., quercetin 3, 7, 3’, 4’-tetrasulphate and quercetin 3-acetyl-7, 3’, 4‘-trisulphate possessed anticoagulant properties. They showed antiplatelet aggregation activity in vitro (28.7 ± 7.8 and 59.9 ± 8.4%) in comparison to the well-known inhibitory quercetin (21.3 ± 6.5%) using a collagen agonist. Quercetin tetrasulphate markedly inhibited the platelet aggregation likely via thromboxane A2 receptor blocking as well as through the inhibition of cyclooxygenase/thromboxane A2 synthetase enzyme. It could be noted that the presence of acetylated residues with lower numbers of sulphate groups markedly inhibited its antiplatelet aggregation activity, with quercetin aglycone structure, which was found pre-requisite for such activities [96]. A study on the semi-synthesis of quercetin-acylated analogues was conducted to improve their lipophilicity. The regioselective quercetin acylation on C3 with variable chains of fatty acids, i.e., quercetin-3-*O*-propionate, quercetin-3-*O*-butyrate, and quercetin-3-*O*-valerate, was tested for their in vitro and in vivo platelet aggregation inhibitions. Results revealed that quercetin propionate and butyrate analogues were more effective than valerate ones, with lipophilicity being a factor controlling biological activity; an equilibrium between the lipophilicity as well as water solubility is still needed to exert antiplatelet aggregation effects [97].

### 7.8. Miscellaneous Activities

#### Wound Healing Activity

Two new acylated flavonoids, isolated from the aerial parts of the fern *Ophioglossum vulgatum* L, were identified as quercetin-3-*O*-[(6-caffeoyl)-*β*-glucopyranosyl(1→3) *α*-rhamnopyranoside]-7-*O*-*α*-rhamnopyranoside (Figure 2-(84)), and kaempferol-3-*O*-[(6-caffeoyl)-*β*-glucopyranosyl (1→3)-*α*-rhamnopyranoside]-7-*O*-*α*-rhamnopyranoside (Figure 2-(85)). Both compounds proved to be active in scratch-wound healing assays on keratinocytes, with kaempferol caffeoyl glycosides being more active, showing an IC_50_ at 198 µM, while quercetin caffeoyl glycoside showed an IC_50_ of 379 µM [98].

## 8. Molecular Docking Study of Selected Biologically Active Compounds in Natural Enzymes

With the aim of highlighting the binding mode of the biologically active compounds with the key amino acids’ residues involved in the binding pockets of the above-mentioned natural enzymes, a molecular docking study was conducted.

The validation of the docking protocol was carried out by redocking the co-crystallized ligand into the active site. A reproduction of the same binding interactions as the co-crystallized ligand demonstrated that the applied docking protocol is suitable for predicting possible binding poses of the compounds. This was also confirmed by the small RMSD values between the poses of the native ligand and the redocked one, as shown in Table 3.

Compounds in (Figure 2-(19,24,25,32 and 33)) that were reported to possess the most potential anticholinesterase activity [66,69,72] were selected to investigate their possible interactions with the binding pocket of the downloaded enzyme. The acetylcholinesterase active site consists mainly of three subsites; a peripheral anionic site (PAS) including Trp286, Tyr124, Asp74, and Phe295; and a mid-aromatic gorge and a catalytic active site (CAS) comprising Trp86, Glu202, Tyr337, and Gly448 residues [99,100]. As shown in Figure 5a, the five compounds were successfully docked in the entire enzymatic gorge with high docking scores ranging from −19.58095 to −27.19648 kcal/mol, Supplementary Table S1. Generally, all compounds bind simultaneously to both the peripheral anionic site (PAS) and catalytic active site (CAS) occupying the entire enzymatic CAS, the PAS, and the mid-gorge tunnel. The compounds were bound to the PAS of the enzyme by establishing hydrophobic interactions with Trp286 and Glu292 and different hydrogen bonding with Tyr72, Asp64, Gly121, Gly122, Tyr124, Ser125, Trp286, Glu292, Ser293, Phe295, and Arg296. Moreover, the compounds were fitted into the CAS exerting important π-π stacking with residues Trp86, Phe338, and Tyr341 in addition to H-bond interactions with Glu202 and Ser203, as shown in Figure 5a and Supplementary Table S1.

Biologically active compounds (Figure 2-(20,21)) were docked in the vicinity of the binding site of butyrylcholinesterase, showing high docking scores, as shown in Supplementary Table S1 and it successfully exhibited key interactions, including π-π stacking with Trp82, hydrogen bonding with the backbone carbonyl group of His438, and hydrogen bonding with three water molecules belonging to the active site of a water network that are firmly anchored to the protein by hydrogen bonds to Asp70, Ser79, and Thr120. Moreover, both compounds exhibited additional H-bond interactions with two amino acid residues Glu197 and Pro285, as shown in Figure 5b.

Upon the docking of compounds (Figure 2-(41,42,43,45 and 46)) in the binding pocket of the downloaded α-amylase co-crystallized with Montbretin A, all compounds interacted with catalytic residues Asp197 and Glu233 via hydrogen bonding interactions. In addition, compound 41 was oriented in the binding site in a manner that allowed for additional hydrogen bonding with the side chain carboxylic acid of Asp300, as shown in Figure 5c. Docking scores of these compounds, as shown in Supplementary Table S1, ranged from −15.8989 to −21.0081 kcal/mol.

Concerning α-glucosidase, it was reported that the main binding of acarbose in the active site involves numerous hydrogen bonds with Asp203, Asp327, Asp542, His600, and Arg526. Consequently, compounds (Figure 2-(47,48,54,55,56,57)) that showed the strongest α-glucosidase inhibition were docked and exhibited two or three hydrogen bonding interactions with the above-mentioned amino acid residues occupying the +1 and-1 sugar subsites, showing high docking scores from −15.6080 to −21.3157 kcal/mol (Figure 5d and Supplementary Table S1).

In the downloaded PDB file of aldose-reductase, the carboxylate group of sulindac, co-crystallized with the enzyme, interacts through H-bonds with Tyr48, His110, and Trp111. It also forms a π–π stacking interaction with the sidechain of Trp20 and has van der Waals contacts with the sidechain of Val47 and Cys298, in addition to the van der Waals contacts between the gating residues Trp111 and Leu300. In the present study, the four most biologically active compounds (Figure 2-(64,65,70,71) were docked in the binding site of aldose reductase and all of them were found to bind to the enzyme with the same interactions mentioned above with docking scores from −15.4179 to −17.3664 kcal/mol, as shown in Figure 5e and Supplementary Table S1.

The HIV integrase inhibitor bound at the active site of the enzyme is involved in several residues forming hydrogen bonds with Glu-152, Thr-66, Lys-159, and Lys-156. Compound 78 was successfully docked in the binding site with a docking score of -16.9625 kcal/mol and it interacted with Gln148, Glu152, and Lys159 through H-bonds. Moreover, the magnesium ion formed another hydrogen bond through close contacts with the carbonyl group of the chromone ring of compound 78 (Figure 5f and Supplementary Table S1).

## 9. Conclusions

This review reports on the potential role and significance of acylated flavonoids. Flavonoids are known to have a variety of biological activities; however, their low bioavailability limited their medicinal usage. Acylated flavonoids are distributed in various plants; moreover, the acylation of flavonoids using hydrolytic and biocatalytic enzymes could be achieved in vitro, in which the synthesis of acylated flavonoids should be carried out with a proper number, position, and type. Taking into consideration that acylated flavonoids showed improved biological activities than their non-acylated analogues, it was deemed of interest to outline recent studies on natural and semi-synthetic acylated flavonoids as promising targets either in pharmaceuticals, food, or cosmetics.

The most common acylated flavonoids found in nature included anthocyanins, flavonoids, viz., quercetin, kaempferol, cyanidin, and peonidin glycosides. It was worth mentioning that anthocyanins acylation could be conjugated either with aromatic moieties on their sugar residues such as “*p*-coumaric, caffeic, sinapic, ferulic and gallic acids” or aliphatic moieties such as “malonic, acetic, malic and oxalic acids”; acylation typically occurs mainly on C3 and less on C6 or C4 of anthocyanins’ sugar structure. In a variety of plants, it was found that acylation with *p*-coumaroyl moiety was more predominant, where diacetylated anthocyanins with *p*-coumaric acid and caffeic acids showed promising biological activities, viz., antioxidant and anti-carcinogenic agents than those acylated with ferulic and hydroxybenzoic acids. Moreover, *p*-coumaroyl acylated flavonoid glucosides were more photostable than their non-acylated glucosides.

In sulphated flavonoids, sulphate moieties can be directly attached to the flavonoid aglycone or to the sugar residue of the glycoside, rendering the entire flavonoid more water soluble and, thus, more biologically active.

Acylated flavonoids proved to exert various biological effects, which were discussed thoroughly during our review, including hepatoprotective, antidiabetic, antiplatelet aggregation, antifungal, and natural enzyme inhibitors. Cyanidin and peonidin glycosides, acylated with caffeic acid, *p*-hydroxy benzoic acid, and ferulic acid residues showed hepatoprotective activities.

Acylated flavonoids can exert an inhibitory effect on several enzymes, which are involved in various metabolic processes in the human body. For example, inhibiting α-amylase and α-glucosidase could be efficient in controlling diabetes; inhibitors of acetyl and butyryl cholinesterases are used for preventing the symptoms of Alzheimer’s disease. The inhibition of the XO enzyme will prevent the breakdown of xanthines to uric acid, as hyperuricemia could be a risk factor of several diseases, whereas the inhibitory action on Na^+^, K^+^ ATPase enzymes provides possible lines of treatment for various cardiovascular disorders.

Acylated caffeoyl glycosides revealed moderate inhibitory action on acetyl cholinesterase enzyme, with higher inhibition effects on butyrylcholinestearse enzymes. High AChE inhibition of C-flavone glycosides was deduced mainly due to the presence of the 4′ methoxy group on ring B and O-glycosylation at C7. Acylated Kaempferol glycosides exhibited strong anti-butyryl choline esterase activity, attributed to the acylation on C3′’ and C6′’ of the sugar residue.

The structural features crucial for the isolated flavonoids’ anti-AD activity related to their antioxidant activities included the presence of C3’ and C4’ hydroxy groups, a C4-oxo group attached to an unsaturated C2-C3 bond, and C3 and C5 hydroxy groups connected to a C4-oxo group. Moreover, quercetin flavonol glycosides were found to possess more neuroprotective potential than kaempferol analogues, and the presence of dihydroxyl groups on ring B and acylation were necessary for enhanced protective actions.

Acylated anthocyanins exhibited more activity on α glucosidase inhibition rather than non-acylated glycosides, assuring the crucial role of the acylated residues of “caffeic or ferulic acid” in the inhibition of α glucosidase activity. In addition, acylated quercetin and kaempferol glycosides revealed moderate inhibitions on α-amylase and stronger action on α-glucosidase enzymes. The caffeoyl esterified flavonoids demonstrated a positive effect on α amylase enzyme inhibition, where the presence of the acyl residue “caffeic acid” within the sugar structure, the hydroxyl groups, and the unsaturated alkyl chain of caffeic acid, was mandatory for anti-diabetic activities, contributing to the promising discovery of natural effective anti-diabetic agents yet requiring further clinical assessments.

Apigenin coumaroyl glucoside expressed potential inhibition on the xanthine oxidase enzyme, and the attachment of the *p*-coumaroyl group to position 2” of the glucose moiety was crucial for activity. Moreover, peonidin glycosides acylated with caffeic, ferulic, and *p*-hydroxy benzoic acid residues inhibited the XO enzyme, where the enzyme’s inhibition was correlated to the presence of hydroxy groups on C5 and C7 of ring A rather than hydroxy groups on ring B.

Galloylated quercetin glycosides exhibited effective inhibition against the (HIV-1) integrase enzyme. The inhibition of such an enzyme could have an efficient role in finding a new drug for the treatment of AIDS disease.

Coumaroyl kaempferol glycosides exhibited inhibitory actions against the Na^+^, K^+^ ATPase enzyme. Inhibitory actions on such an enzyme exerts lines of treatment for various cardiovascular disorders.

In an attempt to increase blood flow, quercetin acetyl trisulphate and quercetin tetra sulphate showed antiplatelet activities; it is noted that the presence of quercetin acetylated residue with a lower number of sulphate groups markedly inhibited its antiplatelet aggregation activity, and it was concluded that there must be a balance between the lipophilicity as well as water solubility in order to exert antiplatelet aggregation effects.

The conducted molecular docking study of the selected biologically active flavonoids in the binding pockets of different natural enzymes, namely acetylcholinesterase, butyrylcholinesterase, α-amylase, α-glucosidase, aldose reductase, and HIV integrase, highlighted the binding modes of these compounds with key amino acids and accordingly justified their mechanisms of action.

Finally, a further elucidation of this interesting class of secondary metabolites could lead to the discovery of new drug, which is of clinical interest.

## Figures and Tables

**Table 3:** Results of the validation of the docking protocol setup.

PDB ID	Enzyme	Co-Crystallized Ligand	RMSD	Docking Score of the Co-Crystallized Ligand (kcal/mol)
4EY7	Acetylcholinesterase	Donepezil	0.8427	−12.7467
4BDS	Butyrylcholinesterase	Tacrine	0.4496	−9.3945
4W93	α-amylase	montbretin A	2.7060	−32.5225
2QMJ	α-glucosidase	Acarbose	0.5016	−26.2699
3RX3	aldose reductase	Sulindac	1.0574	−8.9588
1QS4	HIV 1 integrase	1-(5-chloroindol-3-yl)-3-hydroxy-3-(2H-tetrazol-5-yl)-propenone	0.6840	−10.2199

## Data Availability

Not applicable.

## Supplementary Materials

The following are available online at https://www.mdpi.com/article/10.3390/molecules27175501/s1, Supplementary Table S1: Docking scores of selected compounds.
