# Supplementary material for: Unveiling Natural and Semisynthetic Acylated Flavonoids: Chemistry and Biological Actions in the Context of Molecular Docking
Source: Molecules. 2022 Aug 26;27(17):5501. doi: 10.3390/molecules27175501 (PMC9458193; doi:10.3390/molecules27175501)
Supplement: Supplementary file 1 [file molecules-27-05501-s001.zip › molecules-1879603-supplementary.pdf]

Supplementary Table S1: Docking scores of selected compounds.

Enzyme	Compound	Docking score (kcal/mol)
**Acetylcholinesterase**	**19**	-24.3624134
	**24**	-25.01554
	**25**	-26.59169
	**32**	-19.58095
	**33**	-27.19648
**Butyrylcholinesterase**	**20**	-36.3606
	**21**	-38.1438
**α-amylase**	**41**	-18.0423
	**42**	-16.3629
	**43**	-21.0081
	**45**	-15.8989
	**46**	-16.0820
**α-glucosidase**	**47**	-15.6080
	**48**	-16.2939
	**54**	-17.2090
	**55**	-19.3764
	**56**	-21.2957
	**57**	-21.3157

**aldose reductase**	**64**	**-15.6581**
	**65**	-15.5959
	**70**	-17.3664
	**71**	-15.4179
**HIV integrase**	**78**	-16.9625
